# Stimulus type and duration affect magnitude and evolution of flicker-induced hyperemia measured by laser speckle flowgraphy at the optic disc and peripapillary vessels

**DOI:** 10.1038/s41598-024-57263-z

**Published:** 2024-03-20

**Authors:** Moe H. Aung, Tomas S. Aleman, Arielle S. Garcia, Brendan McGeehan, Gui-Shuang Ying, Robert A. Avery

**Affiliations:** 1 Departments of Neurology, The Perelman School of Medicine at the University of Pennsylvania, Philadelphia, PA USA; 2 Department of Ophthalmology, The Perelman School of Medicine at the University of Pennsylvania, Philadelphia, PA USA; 3 Division of Ophthalmology, The Children’s Hospital of Philadelphia, Philadelphia, PA USA; 4 Department of Ophthalmology, Dell Medical School at The University of Texas at Austin, Austin, TX USA

**Keywords:** Laser speckle flowgraphy, Functional hyperemia, Flicker-induced hyperemia, Neurovascular coupling, Optic nerve head, Neuro-vascular interactions, Visual system

## Abstract

Neurovascular coupling is a vital mechanism employed by the cerebrovascular system, including the eye, to regulate blood flow in periods of neuronal activation. This study aims to investigate if laser speckle flowgraphy (LSFG) can detect coupling response elicited by flickering light stimuli and how variations in stimulus type and duration can affect the magnitude and evolution of blood flow in the optic nerve head (ONH) and peripapillary vessels. Healthy adults were exposed to two types of 10-Hz flicker stimuli: a photopic negative response-like stimulus (PhNR-S) or a visual evoked potential-like stimulus (VEP-S)—each presented in separate 10- and 60-s epochs. Both PhNR-S and VEP-S significantly increased ONH blood flow (p < 0.001) immediately after flicker cessation, with a trend of 60-s stimuli (PhNR-S = 11.6%; VEP-S = 10.4%) producing a larger response than 10-s stimuli (PhNR-S = 7.5%; VEP-S = 6.2%). Moreover, exposure to 60-s stimuli elicited a significantly prolonged ONH hyperemic response, especially with PhNR-S. Lastly, stimulation with either 60-s stimuli elicited a robust increase in blood flow within the peripapillary arterioles (p < 0.01) and venules (p < 0.01) as well. Flicker stimulation with common visual electrophysiology stimuli (PhNR-S and VEP-S) induced a demonstrable increase in ONH and peripapillary vessel blood flow, which varied with flicker duration. Our results validate that LSFG is a robust method to quantify flicker-induced hyperemic responses and to study neurovascular coupling in humans.

## Introduction

Functional hyperemia is a hemodynamic response utilized by the central nervous system, including the inner retina, to modulate local blood flow to match increased metabolic demands. This vascular response is mediated by intricate communications between the neurons, glial cells, and vessels—known as neurovascular coupling^1–4^. Mounting evidence has shown that dysregulation of functional hyperemia may compromise ocular health and contribute to the pathogenesis of different ocular diseases^5–12^. Therefore, reliable and sensitive measurement of inner retinal blood flow can be a valuable tool to better understand the pathogenesis of different ocular diseases and may serve as a biomarker to identify patients at risk or to monitor evolution of disease course.

The hyperemic response has been classically studied by stimulating the eye with flickering light, which maximally activates the inner retina, resulting in increased retinal blood flow^2,3,13,14^. However, assessment of flicker-induced hyperemic response has remained challenging. Previously, many studies have used changes in vessel diameter in response to photic stimulation as the surrogate to changes in retinal blood flow^13,15–18^. Fortunately, new imaging modalities have emerged to overcome previous limitations to provide a more robust and detailed analysis of the retinal hemodynamics^6,15,19–22^. One such promising technique is laser speckle flowgraphy (LSFG), which has garnered much interest as a tool for non-invasive in vivo analysis of ocular perfusion in animal models and humans due to its short acquisition times with high temporal and spatial resolution^23–34^. Two recent studies have demonstrated the capability of LSFG to quantify retinal and optic nerve head (ONH) blood flow during flicker stimulation in human^35,36^. Though these studies showed that custom-built very bright diffuse luminance flicker can reliably lead to enhanced retinal and ONH blood flow, the intensity and frequency of the stimulus is well beyond what humans perceive outside of an experimental setting. To this end, common and clinically used electrophysiology stimuli could provide a more physiologically relevant model of neurovascular coupling along with the potential benefits of targeted stimulation of different retinal cell types as well as easy standardization across institutions. Therefore, the objective of this pilot study is to determine if commonly used electrophysiology light stimuli—specifically photopic negative response stimulus and visual evoked potential stimulus at more comfortable light intensity—can elicit increased inner retinal blood flow detectable by LSFG. Moreover, we are interested in examining how alterations in the flicker stimulus duration can affect the morphology and evolution of hemodynamic response in the human eye.

## Material and methods

### Subjects

This study protocol was approved by the Children’s Hospital of Philadelphia Institutional Board of Review. Written informed consent was obtained from all participants after explanation of the study purpose and protocols and before initiation of the experiment. All methods in this study were performed in accordance with the relevant guidelines and regulations. All data was collected and stored according to HIPAA guidelines.

Healthy subjects were recruited for the study and were eligible to complete the study if they had best-corrected visual acuity of at least 20/20 and a normal ophthalmic examination including measurement of intraocular pressure (IOP), color vision, slit lamp examination, and direct fundoscopy. Subjects were also screened for any vascular diseases that may alter the hemodynamics of the eye, such as diabetes, hypertension, or hyperlipidemia. Other exclusion criteria included age less than 18 years, history of ophthalmic diseases, history of vascular interventions (for example thrombectomy, cardiac stent placement, cerebral angiogram), history of epilepsy or migraine headache that may increase sensitivity to flickering light, pregnancy or lactation, and smoking.

All subjects were asked to abstain from alcoholic and caffeinated beverages on the day of the study (with at least 4 h gap) to minimize potential confounding influence on IOP and/or blood pressure. Thirty minutes before the experiment, baseline blood pressure and heart rate measurements for each subject were taken. Also, both pupils of each subject were dilated with 0.4% tropicamide (Mydriacyl 0.4%; Alcon Laboratories, Fort Worth, Texas, USA), but testing was only done on the right eye of each subject.

### Flicker stimulation experimental paradigm

**Figure 1:**
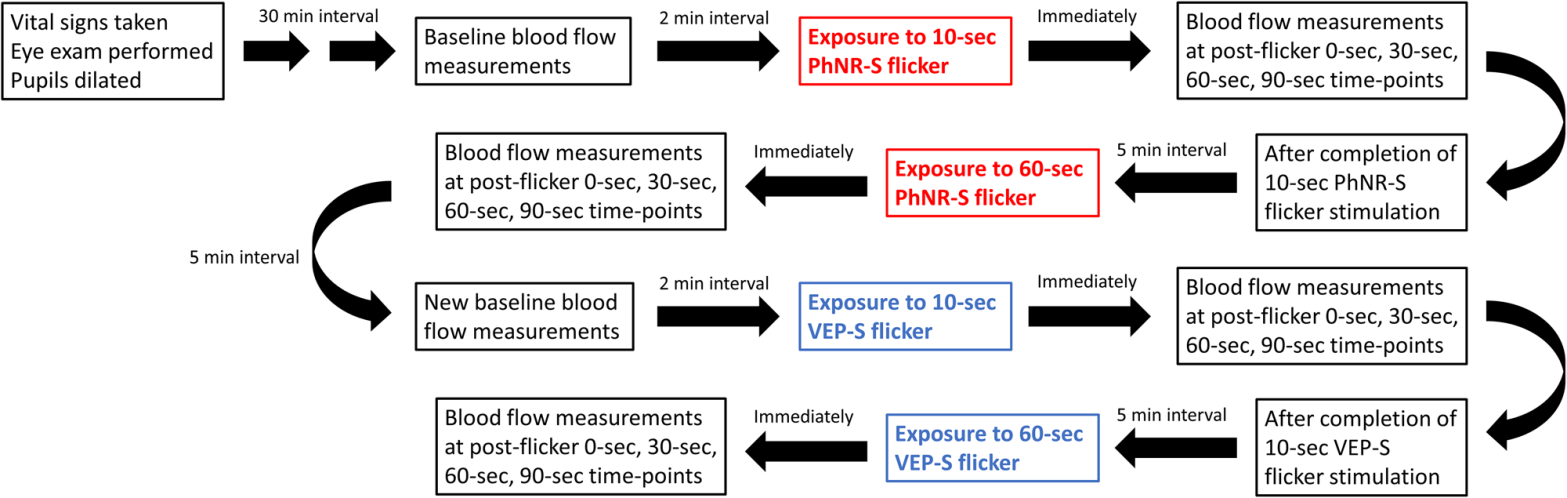
Diagram illustrating the procedures for flow of the experiments in the right eye of each subject. Of note, two replicates of blood flow measurements were taken at each baseline (before exposure to PhNR-S or VEP-S), while blood flow measurement was taken once at different time-points after cessation of flicker stimulation.

After the pupils were adequately pharmacologically dilated, a set of baseline measurements (two replicates per measurement) of the blood flow at the ONH and surrounding retinal vessels from right eye was measured under ambient room light condition (Fig. 1). After taking baseline measurements, the right eye of each subject was then exposed to a full-field photopic negative response-like light stimulus (PhNR-S), which is a 640 nm red light (3 cd s/m^2^) on a 465 nm blue background (12.5 cd s/m^2^). The stimulus was presented at 10 Hz for 10 s. Blood flow at the ONH and peripapillary retinal vessels were serially measured at 0-s, 30-s, 60-s, and 90-s time-points after cessation of the flicker stimulation. After completion of the 10-s PhNR-S flicker stimulation, there was a 5-min break before exposure of another flicker stimulation to allow for recovery of the ocular blood flow to baseline. The right eye of each subject was then exposed to 60 s of PhNR-S flickering at 10 Hz. Identical serial measurements of the blood flow at the ONH and retinal vessels were made.

After completion of the PhNR-S flicker stimulation experiment, there was a resting period of 5 min. Afterward, a new set of baseline measurements (two replicates per measurement) for the VEP-S flicker stimulation experiment was taken. The right eye of each subject was then exposed to a full-field visual evoked potential-like light stimulus (VEP-S), which is a diffuse white light (3 cd s/m^2^) presented at 10 Hz for 10-s. Immediately after cessation of flicker stimulation, serial measures of blood flow at the ONH and retinal vessels were taken, similar to the measuring protocol after PhNR-S flicker stimulation as described above. After another resting interval of 5 min, the right eye of each subject was exposed to 60 s of VEP-S stimulus flickering at 10 Hz, followed by serial measures of blood flow at the ONH and retinal vessels. Resting interval of 5 min in-between stimulation session was chosen based on prior literature of works showing that flicker-induced hyperemic response typically resolved within 1–2 min after cessation of flicker exposure^3^.

### Laser speckle flowgraphy

Blood flow at the ONH and peripapillary retinal vessels was assessed using a commercially available LSFG system (LSFG-NAVI; Softcare Co. Ltd, Fukuoka, Japan). The principles and set-up of LSFG have been outlined in length previously^27,29,32^. In brief, the instrument is comprised of a fundus camera equipped with an 830 nm diode laser as the light source and a digitally charged-coupled device camera (750 × 360 pixels) as the detector. After illuminating the ocular fundus with the laser light source, a speckle contrast pattern is produced by the scattering and interference of the illumination due to the moving erythrocytes. By examining the changes in the speckle contrast pattern, the built-in software (LSFG Analyzer; Softcare Co. Ltd, Fukuoka, Japan) determines the mean blur rate (MBR), which is a quantitative index of blood flow (expressed in arbitrary units). During a single LSFG scan, a total of 118 images are acquired at a rate of 30 frames per seconds over a 4-s period. The software then synchronizes and averages all acquired images into a single scan to produce a “composite map” showing the perfusion pattern in the examined ocular fundus.

### Assessment of optic nerve head blood flow

**Figure 2:**
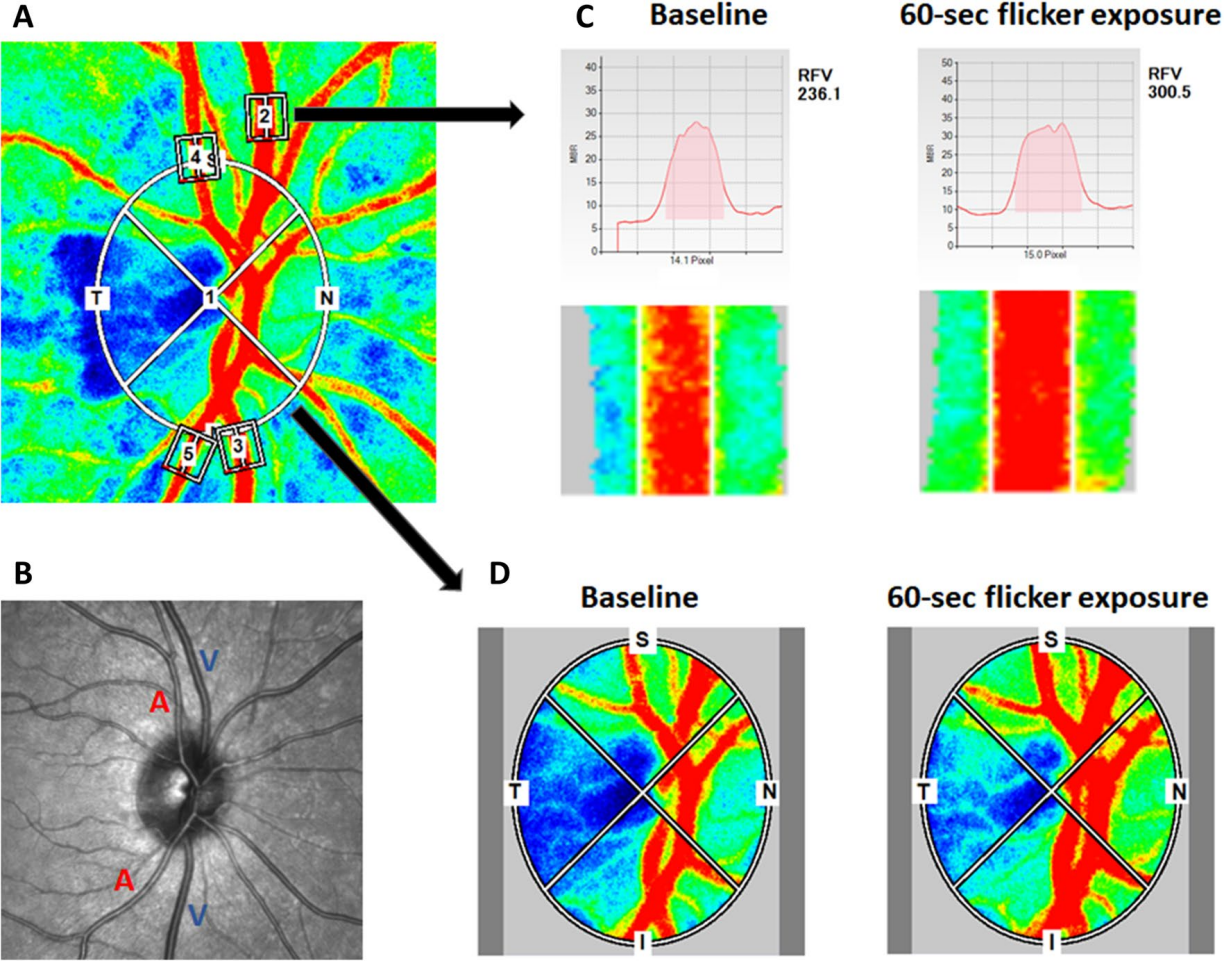
Diagram illustrating how blood flow at different regions of interest (ROI) increased with flicker stimulation. (**A**) A representative LSFG composite map taken from one of the subjects showing different ROIs: 1—the overall optic nerve head, 2—a superior venule, 3—an inferior venule, 4—a superior arteriole, and 5—an inferior arteriole. (**B**) This is the corresponding infrared image of the fundus marking the selected arterioles and venules. (**C**) A graphical illustration of how the built-in software (LSFG Analyzer) calculates the relative flow volume of a selected ROI at baseline and at 0-s time-point after flicker cessation. (**D**) A graphical illustration of how the built-in software (LSFG Analyzer) isolates the mean blur rate of the optic disc at baseline and at 0-s time-point after flicker cessation.

For analysis of the ONH blood flow in response to flicker stimulation, an ellipsoid region of interest was first positioned manually to cover the optic disc on the color-coded fundus map using the LSFG Analyzer software (Fig. 2). The LSFG Analyzer software then subtracts the MBR value of the tissue areas within the optic disc from the overall MBR value of the entire optic disc, and thus yielding the blood flow within the ONH^27,35^. As MBR is calculated in arbitrary units and cannot be used to make comparisons between different eyes/subjects, percent change of the ONH blood flow for each eye at different time-points was calculated in relation to the eye’s corresponding baseline ONH blood flow.

### Assessment of peripapillary vessel blood flow

For analysis of blood flow within peripapillary retinal vessels in response to flicker stimulation, four vessels total were selected for each eye: two arterioles (one superior and one inferior) along with two corresponding venules (one superior and one inferior). Rectangular region of interest was placed around each selected vessel near the rim of the optic disc (Fig. 2). Blood flow within the vessel at the chosen region of interest is then determined by measuring relative flow volume (RFV) as detailed previously^29,32^. Briefly, the MBR values for retinal vessels always include the background intensity of choroidal blood flow. Therefore, LSFG Analyzer software can calculate the retinal blood flow by subtracting background choroidal MBR from overall MBR of the region of interest, and thus yielding RFV value of the vessel. Again, percent changes of average RFV for each vessel from each eye at different time-points was calculated in relation to the eye’s corresponding baseline RFV of that particular vessel.

### Statistical analysis

For each measure of ONH blood flow and peripapillary vessel blood flow, the percentage change from baseline (before flicker stimulation) was calculated at each subsequent time-point (0, 30, 60, 90 s) after cessation of stimulation under each experimental condition. Descriptive analyses were performed for each measure of ONH blood flow and peripapillary vessel blood flow using mean, standard deviation (SD), and standard error of mean (SEM). At baseline, the variation between two replicated measures was calculated as the percent difference between two repeated measures, and their averaged value was used for calculating of percent change from baseline for each time point after cessation of stimulation. For statistical comparisons, mixed-effects models were used to compare the percentage change over time in blood flow with random intercept for each eye. For each blood flow measure, three mixed-effects models were created: (1) a model including only the main effects for time, stimulus type, and stimulus duration; (2) a model of main effects and all of their two-way interactions; and (3) a model of main effects, their two-way interactions and their three-way interactions. Each model also included an additional covariate controlling for the percentage change of heart rate at each time-point. Heart rate measurements used at each time-point of blood flow assessment are computed internally by the LSFG Analyzer software. These heart rate measurements are different from the initial heart rate measurement (thirty minutes before start of the experiment) that was taken using pulse oximeter at fingertip.

Mean and standard error estimates for percent change from baseline at each time-point were derived from the main effects model and tested against a null hypothesis value of 0, to determine whether there is statistically significant change from the baseline. The models with interaction terms were used to test whether the percent change over time in blood flow measures differs between stimulus type, and stimulation duration. If no significant interactions were found, the statistical comparisons were based on the models with main effects only. All statistics analyses were performed in R version 4.03, and two-sided p < 0.05 was considered to be statistically significant.

## Results

A total of ten adults (6 females and 4 males) met all inclusion and exclusion criteria and were included in the study. The mean age (± SD) was 31 ± 3 years (range 19 to 48 years). The mean baseline arterial pressure (MAP) was 89 ± 4 mmHg, and mean heart rate was 79 ± 8 beats per minute. Mean IOP of study eye (one study eye per subject) was 16 ± 1 mmHg. All blood flow changes in response to different flicker stimulations at the ONH and peripapillary arterioles and venules are summarized in Table 1.

**Table 1:** Summary of all the blood flow changes at the ONH and peripapillary vessels in response to different flicker stimulation paradigms explored in this study.

Type of stimulus	0-s time-point	30-s time-point	60-s time-point	90-s time-point
Changes in overall ONH blood flow				
PhNR-S 10-s	7.46 ± 3.26	2.81 ± 1.85	− 0.84 ± 3.03	2.05 ± 2.12
PhNR-S 60-s	11.64 ± 2.28	7.90 ± 3.95	5.99 ± 3.38	3.96 ± 2.16
VEP-S 10-s	6.21 ± 1.94	1.08 ± 1.86	0.38 ± 1.87	3.66 ± 1.60
VEP-S 60-s	10.38 ± 3.14	4.05 ± 3.11	1.73 ± 2.51	1.86 ± 3.65
Changes in superior arteriolar blood flow				
PhNR-S 10-s	2.55 ± 5.40	3.62 ± 4.42	3.59 ± 5.49	7.96 ± 3.16
PhNR-S 60-s	11.37 ± 2.36	0.51 ± 3.65	5.44 ± 3.85	5.95 ± 5.62
VEP-S 10-s	8.54 ± 2.53	− 2.36 ± 1.97	5.15 ± 2.94	8.88 ± 2.65
VEP-S 60-s	11.73 ± 5.53	4.45 ± 5.29	3.31 ± 5.11	5.12 ± 5.03
Changes in inferior arteriolar blood flow				
PhNR-S 10-s	− 0.17 ± 2.56	− 2.64 ± 3.06	− 6.53 ± 3.90	0.65 ± 1.98
PhNR-S 60-s	6.46 ± 3.68	7.13 ± 3.66	2.07 ± 3.47	1.41 ± 1.97
VEP-S 10-s	8.24 ± 3.02	2.92 ± 2.59	3.82 ± 3.19	4.94 ± 2.95
VEP-S 60-s	10.85 ± 6.76	4.10 ± 2.96	2.32 ± 3.53	3.99 ± 4.36
Changes in superior venular blood flow				
PhNR-S 10-s	3.96 ± 4.27	− 0.16 ± 3.78	0.30 ± 4.43	8.43 ± 3.89
PhNR-S 60-s	13.48 ± 3.78	− 0.25 ± 3.80	2.50 ± 4.86	7.08 ± 5.00
VEP-S 10-s	11.43 ± 2.45	3.12 ± 2.35	3.04 ± 2.19	5.94 ± 2.43
VEP-S 60-s	15.68 ± 6.05	7.13 ± 4.80	5.77 ± 4.78	2.95 ± 5.20
Changes in inferior venular blood flow				
PhNR-S 10-s	3.90 ± 2.36	− 2.90 ± 1.93	− 3.75 ± 2.43	0.47 ± 3.55
PhNR-S 60-s	7.64 ± 3.84	4.51 ± 5.54	2.50 ± 3.08	3.78 ± 3.28
VEP-S 10-s	4.07 ± 2.81	− 1.62 ± 2.60	0.97 ± 2.44	4.27 ± 3.05
VEP-S 60-s	11.24 ± 4.76	0.44 ± 3.60	− 0.81 ± 2.45	0.99 ± 3.96

Data presented here are mean ± SEM.

### Changes in optic nerve head blood flow after flicker stimulation

**Figure 3:**
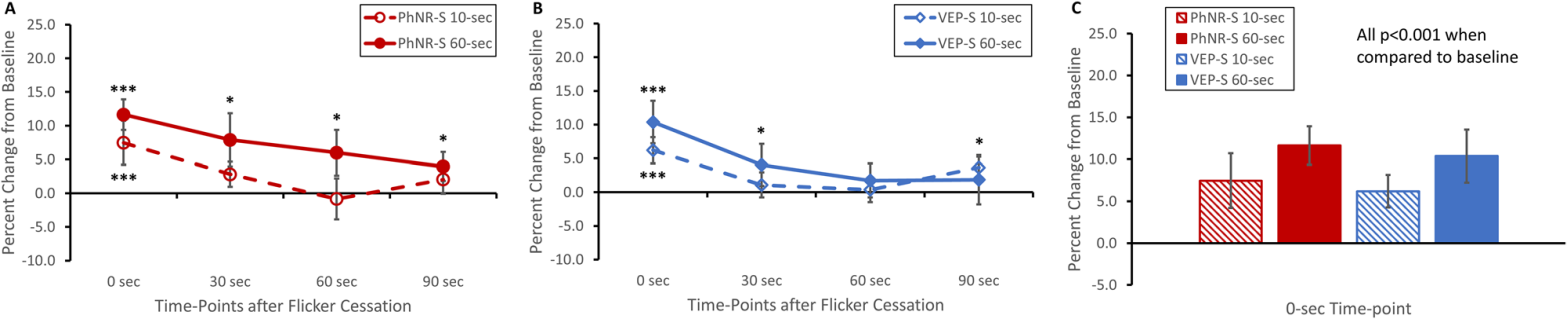
Changes in overall ONH blood flow in response to (**A**) PhNR-S or (**B**) VEP-S over time. Across all time-points after cessation of stimulation (0-, 30-, 60-, 90-s time-points), 60-s stimuli elicited a larger response than that of 10-s stimuli in both (**A**) PhNR-S and (**B**) VEP-S experiments. When looking at 0-s time-point immediately after cessation of flicker stimulation (**C**), there was a significant increase in ONH blood flow in response to each stimulus type and stimulation duration, with trend of large augmentation with longer stimulus duration. Exposure to 60-s PhNR-S resulted in persistently elevated blood flow throughout the studied time-points. Of note, baseline replicate measure variations for ONH blood flow were the following (± SEM): PhNR-S − 4.1% ± 3.6 and VEP-S 0.6% ± 2.7. All data plotted in the figure are mean ± SEM. Definitions for the asterisks are the following: *p < 0.05, **p < 0.01, ***p < 0.001.

Upon cessation of flicker stimulation and immediate measurement using the LSFG device (0-s time-point), blood flow of the entire optic disc region significantly increased when comparing to baseline (i.e. before flicker stimulation), regardless of stimulus type or duration (Fig. 3A–C: p < 0.001). Though not statistically significant, there was a trend of larger magnitude of ONH blood flow increase with longer stimulus duration immediately after flicker stimulation (Fig. 3C: 10-s PhNR-S 7.5% to 60-s PhNR-S 11.6% and 10-s VEP-S 6.2% to 60-s VEP-S 10.4%). This flicker-induced blood flow augmentation dissipated with return to baseline for both 10-s PhNR-S and VEP-S, starting at 30-s time-point and onward (Fig. 3A,B). On the other hand, the blood flow changes induced by 60-s flickering VEP-S resolved at later time-point: 60-s time-point (Fig. 3B). Though there was a gradual decline in the hyperemic response induced by 60-s PhNR-S flicker, the hyperemic response was significantly sustained throughout the studied time course (Fig. 3A: p < 0.05 for all time-points). There was also a significant overall main effect of stimulus duration on changes in ONH blood flow such that 60-s flickering stimuli resulted in higher increase in ONH blood flow than that of 10-s flickering stimuli (Fig. 3: p = 0.01). These findings were consistent when evaluating the ONH blood flow by quadrants as well: superiorly, inferiorly, temporally, or nasally (data not shown).

### Changes in arteriolar blood flow after flicker stimulation

**Figure 4:**
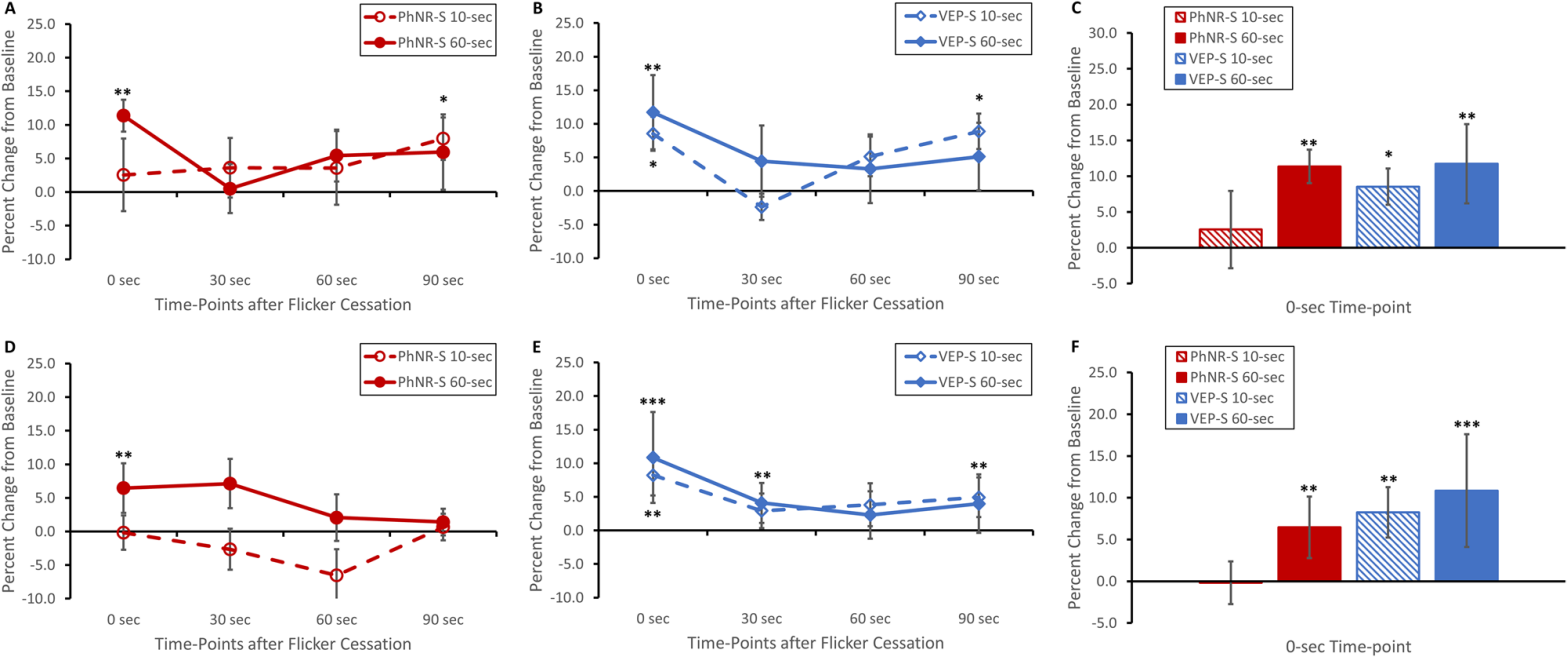
Changes in blood flow over time after flicker stimulation for (**A**–**C**) superior and (**D**–**F**) inferior arterioles. (**A**–**C**) For superior arteriolar blood flow, both 10-s and 60-s VEP-S elicited significantly increased vascular blood flow at 0-s time point after flicker stimulation when compared to baseline (i.e. before stimulation), while only exposure to 60-s PhNR-S resulted in significant blood flow increase. The effect of flick stimulation resolved at 30-s time-point. However, there was an intriguing rebound increase in blood flow at 90-s time-point after exposure to either 60-s PhNR-S and VEP-S stimuli. Of note, baseline replicate measure variations for superior arteriolar blood flow were the following (mean ± SEM): PhNR-S − 10.9% ± 4.5 and VEP-S 3.9% ± 4.7. (**D**–**F**) As for the inferior arterioles, both 10-s and 60-s VEP-S as well as 60-s PhNR-S resulted in significant hyperemic response. Similar to superior arterioles, the effect of flick stimulation on inferior arterioles dissipated at 30-s time-point for almost all the stimuli (except 60-s VEP-S). Exposure to 60-s VEP-S resulted in persistently elevated blood flow when compared to baseline at 30-s and 90-s time-points. On the other hand, PhNR-S 10-s did not lead to any blood flow increase, but instead a trend of decreasing blood flow that peaked at 60-s time-point. Of note, baseline replicate measure variations for inferior arteriolar blood flow were the following (± SEM): PhNR-S − 5.7% ± 4.4 and VEP-S − 2.3% ± 4.0. All data plotted in the figure are mean ± SEM. Definitions for the asterisks are the following: *p < 0.05, **p < 0.01, ***p < 0.001.

For superior arcuate arterioles, both 60-s PhNR-S and VEP-S resulted in significant hyperemic response at 0-s time-point immediately after flicker stimulation (Fig. 4A–C: p < 0.01 for both stimuli). As for 10-s stimuli, only VEP-S elicited a significant arteriolar blood flow increase (Fig. 4A–C: p < 0.05). In terms of progression of blood flow changes, the effect of all flicker stimulation resolved by 30-s time-point with the superior arteriolar blood flow being similar to that of baseline (Fig. 4A,B). Interestingly, there was a secondary increase in blood flow at 90-s time-point after exposure to 60-s flicker stimulus, regardless of stimulus type (Fig. 4A,B: p < 0.05).

For inferior arcuate arterioles, both 10-s and 60-s VEP-S resulted in significant hyperemic response at 0-s time-point immediately after flicker stimulation (Fig. 4E,F: p = 0.003 for 10-s VEP-S and p < 0.001 for 60-s VEP-S). As for PhNR-S flicker, only the 60-s duration caused a significant hyperemic response at 0-s time-point (Fig. 4D,F: p = 0.008). For 60-s PhNR-S and 10-s VEP-S, the inferior arteriolar blood flow returned to baseline at 30-s time-point (Fig. 4D,E). On the other hand, the hyperemic response elicited by 60-s VEP-S dissipated at 60-s time-point with a secondary rebound elevation at 90-s time-point (Fig. 4E: p = 0.005). There was also a significant main stimulus type effect with VEP-S causing overall larger inferior arteriolar blood flow changes than PhNR-S (Fig. 4D–F: p = 0.02).

### Changes in venular blood flow after flicker stimulation

**Figure 5:**
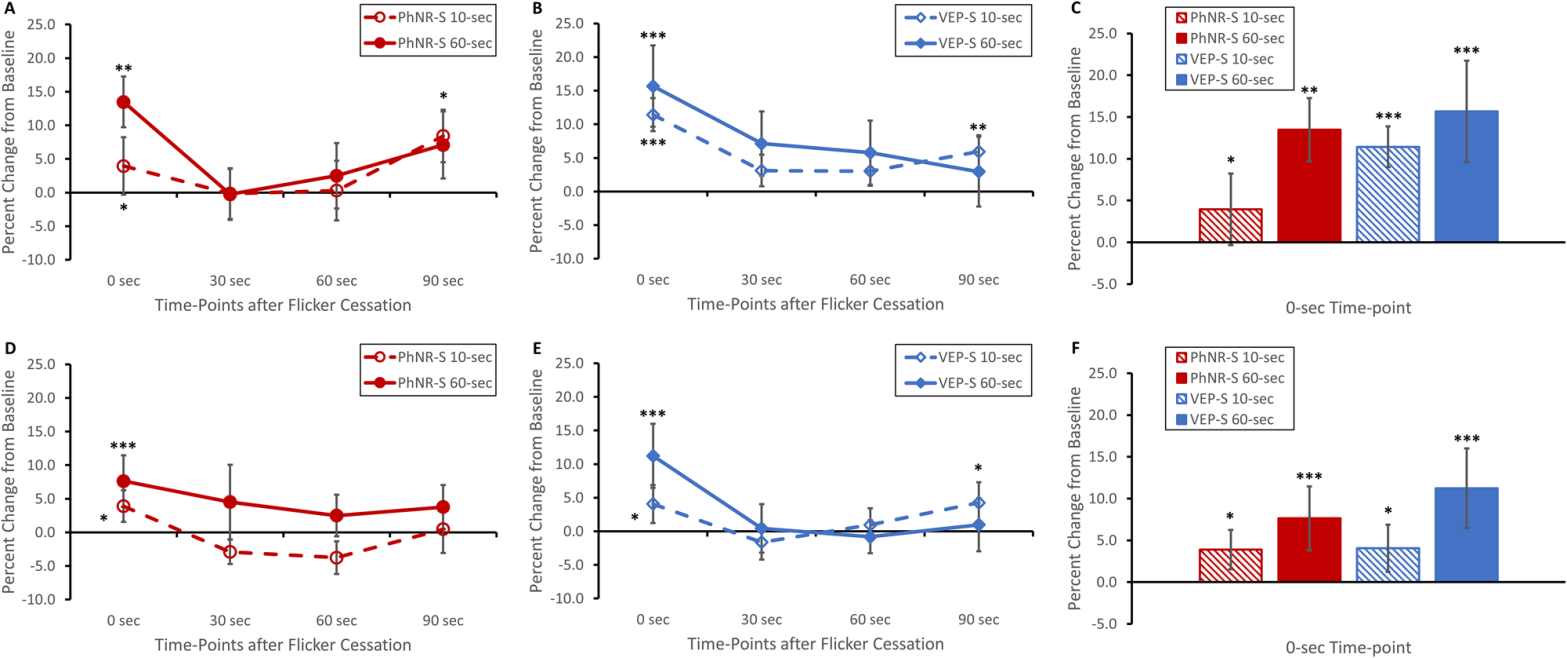
Changes in blood flow over time after flicker stimulation for (**A**–**C**) superior and (**D**–**F**) inferior venules. (**A**–**C**) For superior venules, there was significant increase in blood flow in response to flicker stimulation (either PhNR-S or VEP-S) at 0-s time-point, with trend of large augmentation with longer stimulus duration. The effect of flick stimulation resolved at 30-s time-point with a rebound increase in blood flow at 90-s time-point after exposure to 60-s PhNR-S, 10-s VEP-S, or 60-s VEP-S. Of note, baseline replicate measure variations for superior venular blood flow were the following (± SEM): PhNR-S − 6.2% ± 3.8 and VEP-S − 2.1% ± 3.6. (**D**–**F**) As for the inferior venules, there was significant hyperemic response to flicker stimulation (either PhNR-S or VEP-S) at 0-s time-point, again with trend of greater increase with longer stimulus duration. Similar to superior venules, the effect of flick stimulation on inferior venules dissipated at 30-s time-point for all the stimuli. However, the rebound increase in blood flow at 90-s time-point only occurred after exposure to 60-s VEP-S. Of note, baseline replicate measure variations for inferior venular blood flow were the following: PhNR-S − 3.4% ± 4.6 and VEP-S − 4.0% ± 2.4. All data plotted in the figure are mean ± SEM. Definitions for the asterisks are the following: *p < 0.05, **p < 0.01, ***p < 0.001.

For superior venular blood flow, exposure to both PhNR-S and VEP-S resulted in significant hyperemic responses at 0-s time-point immediately after flicker stimulation (Fig. 5A–C: p < 0.05 for 10-s PhNR-S, p < 0.01 for 60-s PhNR-S, and p < 0.001 for both 10-s and 60-s VEP-S). The evolution of the flicker-induced superior venular responses was similar to arteriolar blood flow, such that the venular blood flow returned to baseline at 30-s time-point for all stimuli, with a secondary blood flow increase at 90-s time-point for 10-s VEP-S, 60-s VEP-S, and 60-s PhNR-S in the absence of new flicker stimulus (Fig. 5A,B: p < 0.05).

Changes in inferior venular blood flow were similar to those of superior venular blood flow. Exposure to PhNR-S and VEP-S resulted in significant hyperemic responses at 0-s time-point immediately after flicker stimulation (Fig. 5D–F: p < 0.05 for 10-s PhNR-S and 10-s VEP-S, p < 0.001 for 60-s PhNR-S and 60-s VEP-S). Again, the flicker-induced hyperemic responses resolved at 30-s time-point for all stimuli (Fig. 5D,E) with a rebound blood flow elevation at 90-s time-point for only 60-s VEP-S (Fig. 5E: p < 0.05). Lastly, there was a significant main effect of stimulus duration in the inferior venular arcade such that 60-s stimuli had larger response compared to 10-s stimuli (Fig. 5D–F: p = 0.03).

## Discussion

Using two common electrophysiology stimulus types presented at two different durations, our study demonstrated that we could rapidly increase ONH blood flow as well as arteriolar and venular blood flow along the arcades. We also showed that these flicker-induced hyperemic response can be reliably measured by the new imaging modality LSFG.

Our study highlights some important factors to consider when interrogating neurovascular coupling using flicker stimulation. First, we used two stimuli with very different properties. The PhNR-S (640 nm red light on a 465 nm blue background) is designed to more directly stimulate cone-driven retinal ganglion cells (RGCs) whereas the diffuse white stimulus for VEP-S is directed at stimulating the entire retina. Interestingly, our results showed that both PhNR-S and VEP-S generated similar magnitude of hyperemic responses at the ONH and at peripapillary vessels, especially when exposed at 60-s duration. We suspect that the relatively high flicker frequency used in this study may potentially underlie the similar responses to either stimuli as such frequency will predominantly stimulate the magnocellular visual pathway^37,38^. Thus, it will be interesting to examine if the flicker-induced hyperemic response to PhNR-S and VEP-S would differ more when these two stimuli are given at lower flickering frequency. Nonetheless, we did find a few trends of differences in blood flow changes between PhNR-S and VEP-S flicker stimulation: (1) 60-s PhNR-S appears to produce a more sustained increase in ONH blood flow over time when compared to VEP-S (Fig. 3) and (2) VEP-S at shorter flicking duration (i.e. 10 s) tends to elicit more consistent vascular changes in the peripapillary vessels when compared to PhNR. Future research with larger sample is first needed to determine if these trends are reproducible. Altogether, results from our current study will add to the body of prior extensive work in evaluating the effects of modifying stimulus parameters (such as flicker frequency, wavelength of the flicker, and color ratio of the flicker) on flicker-induced hyperemic response^3,37–40^.

Another important factor to consider in generating robust hyperemic response is the duration of stimulus presentation. In accordance with prior study that measured changes in retinal vessel diameter to flicker stimulation as a surrogate for functional hyperemia, our current study found that longer duration of stimulation yields a trend of larger and more consistent hyperemic response^41^. Specifically, we found that when either stimulus (PhNR-S or VEP-S) was presented for 10-s, ONH blood flow increased for ~ 30 s before returning to baseline. On the other hand, 60-s flicker stimuli led to a significantly increased ocular blood flow that was sustained longer without returning to baseline till 60-s time-point for VEP-S and beyond 90-s time-point for PhNR-S. This prolonged increase in ONH blood flow after longer duration of flicker stimulation is also consistent with prior animals studies in which post-flicker ONH blood flow gradually returned to baseline after 2–3 min^33,42^. In contrast, hyperemic responses in both arterioles and venules along the arcades dissipated quickly, mostly before our 30-s measurement time-point. Such evolution of the post-flicker blood flow changes in the vessels also aligns with prior studies of functional hyperemia in human using changes in retinal vessel caliber as a surrogate for hemodynamic changes. In those studies, the vascular width returned to baseline rapidly after flicker cessation, usually within 10–20 s^2,3,13^.

In addition, when evaluating blood flow changes in peripapillary vessels in response to flicker stimulation, we noticed a rebound blood flow increase at 90-s time-point after the blood flow appeared to fall back to baseline at 60-s time-point. To determine if this observation is a true effect or simply an artifact, further studies with shorter measurement intervals (every 15-s measurement rather than current 30-s measurement) and longer total measurement duration (measuring up to 3–5 min instead of current 1.5 min) after flicker cessation are warranted. Also, it will be intriguing to assess if each subject’s systemic blood pressure may have varied at each time-point of LSFG measurement, and thereby serving as potential contributing/confounding factor.

Differences in imaging techniques and flicker stimuli must be considered when comparing our findings to other human studies using LSFG. In 2018, Fondi and colleagues showed that LSFG can reliably quantify changes in ONH blood flow and individual retinal arteries and veins in response to flicker stimulation in human subjects^35^. The magnitude of the ONH hyperemic responses found in our studies peaked at 11–15%, while Fondi’s study found increased ONH blood flow in the range of 17–29%. However, in a more recent study that examined ONH blood flow changes in response to diffuse white illuminance flicker (at 12 Hz, 9000 cd/m^2^) amongst normal tension glaucoma and healthy individuals, Mursch-Edlmayr et al. reported the increases in blood flow to be approximately 1.5% to 7.9% after 1 min of stimulation (which are lower than reported in our study)^36^. We suspect that differences between flicker stimulation paradigms, specifically the brighter flicker luminance (9000 to 10,000 cd/m^2^) and slightly higher flicker frequency (12 Hz) provided in these studies, likely explain the discrepancies. Furthermore, their light source was attached to the camera, and thus permitting instant measurement of the ONH blood flow after flicker exposure. On the other hand, our current paradigm required the subject to be quickly repositioned back in front of the LSFG device for imaging after each flicker stimulation, creating a short delay that may result in our inability to capture the maximal flicker-induced hyperemic response. Therefore, future improvement in our setup may help us minimize potential variabilities or fluctuations in the hyperemic responses when assessed with LSFG.

Other limitations should be considered when interpreting our study results. Similar to other imaging devices, the LSFG calculates blood flow using arbitrary units, and thus complicating a direct comparison between patients and forcing us to rely on a within subject change^27,29,32^. However, by evaluating the percent change in MBR or RFV (as illustrated in this study) from baseline/resting state to post-flicker time-point(s), we were able to reliably measure within subject changes. In addition, as eloquently described by Fondi and his colleagues, LSFG analysis software attempts to account for the impact of background choroid blood flow, but some influence cannot be ruled out^35^.

In summary, our current work highlights LSFG as a promising non-invasive imaging modality to study neurovascular coupling by examining flicker-evoked hyperemic responses in human peripapillary vasculature as well as the optic nerve head. It is essential to consider differences in stimulus type and stimulus duration when monitoring the temporal course of ocular blood flow in humans using flicker provocation. With the protocols established in this study, we hope to evaluate and compare how different pathologies (such as ischemia, inflammation, infection) of the optic nerve affect the hyperemic responses in the near future. Ultimately, investigating the neurovascular coupling response under diseased state may provide critical insight into to the development, assessment, and treatment of various ocular disorders.

## Data Availability

The datasets generated from the current study are available from the corresponding author on reasonable request.
